# Evaluating the current state of Mendelian randomization studies: a protocol for a systematic review on methodological and clinical aspects using neurodegenerative disorders as outcome

**DOI:** 10.1186/s13643-018-0809-3

**Published:** 2018-09-24

**Authors:** Sandeep Grover, Fabiola Del Greco M., Inke R. König

**Affiliations:** 1 Institut für Medizinische Biometrie und Statistik, Universität zu Lübeck, Universitätsklinikum Schleswig-Holstein, Campus Lübeck, Lübeck, Germany; 2 Institute for Biomedicine, EURAC Research, Via Galvani 31, Bolzano, Italy

**Keywords:** Mendelian randomization, Instrumental variable, Observational epidemiology, Genome-wide association study, Summary data, Pleiotropy, Casual inference

## Abstract

**Background:**

Mendelian randomization (MR) is fast becoming a popular method to judge causality from routinely conducted observational studies. However, stringent underlying statistical assumptions, missing biological information, and high sample size requirement might make it prone to misuse. Furthermore, rapidly updating methodologies and increasingly available datasets to researchers are making the interpretations of heterogeneous results even more complicated. In this protocol, we provide our design for a multifaceted systematic review on MR studies using neurodegenerative disease as an example outcome. The planned systematic review which has already passed the pilot stage will help to develop an in-depth understanding of how various MR methods have been applied, what has been achieved, and what can be done in future for to arrive at true causal risk factors.

**Methods:**

During the pilot phase of this systematic review, several versions of questionnaires and frequent consultations between reviewers helped us to finalize a comprehensive list of questions. This will be used to extract information on systematically searched MR articles investigating causality underlying neurodegenerative diseases. A literature search of the electronic databases (Embase, MEDLINE, Web of Science, Scopus, and databases listed in the Cochrane library) will be conducted. The search strategy will include terms related to MR and the spectrum of neurodegenerative diseases. Two independent reviewers will screen the studies, and three will extract the data. The included studies will be further judged by two reviewers for accuracy and completeness of available information. We will perform descriptive and quantitative synthesis using sensitivity analyses of causal association by study design, selection of genetic instrument, validity of MR assumptions, MR method, and sensitivity analysis based on exclusion of potential pleiotropic variants. The quality of conduct as well as quality of reporting in the included studies will be assessed and reported. A meta-analysis will be conducted, if effect estimates on identical genetic instruments are available for both exposure and outcome in the studies using data from participants from ethnically similar populations.

**Discussion:**

This systematic review protocol utilizes a unique comprehensive data abstraction tool based on recent methodological advancements in the field of MR. The planned systematic review will further integrate information on methodological details with clinical findings in latest available large-scale genome-wide association study datasets. Our findings aim to help raising awareness and promoting transparent reporting of MR studies.

**Systematic review registration:**

PROSPERO CRD42018091434.

**Electronic supplementary material:**

The online version of this article (10.1186/s13643-018-0809-3) contains supplementary material, which is available to authorized users.

## Introduction

Neurodegeneration, which occurs in diseases such as amyotrophic lateral sclerosis (ALS), Alzheimer’s disease (AD), multiple sclerosis (MS), and Parkinson’s disease (PD), is characterized by diverse pathological conditions and heterogeneous clinical presentations. These diseases are slow progressing with late visible phenotypic characteristics and are often untreatable [1]. Most of our current knowledge on the etiology of neurodegenerative diseases comes from observational studies which suggest an underlying multifactorial etiology due to complex gene-environment interaction in patients suffering from non-monogenic disease subtypes [2]. Identification of both causal environmental risk factors and genetic predisposing factors could be central to developing preventive or early therapeutic approaches. Environmental risk factors for neurodegenerative diseases in general show small effect sizes which make meaningful causal interpretation in the presence of the underlying known and unknown confounding in observational studies extremely difficult. For instance, a recent comprehensive systematic review identified pesticides and head injury as the most frequently reported potential risk factors for PD [2]. On the other hand, common environmental factors such as smoking, alcohol, and coffee were reported as potential protective factors with most convincing evidence based on meta-analysis of observational studies. Another challenge in interpreting causality from observational studies is the long pre-clinical period of neurodegenerative diseases as it becomes difficult to conclude whether a potential risk factor actually triggered the disease or an abnormal level of risk factor was the result of early undetectable phase of the neurodegenerative disease. Although randomized controlled trials (RCTs) slowly and gradually have been adding to our previous understanding of the effect of treatment on disease symptoms, most of the desired hypothesis including those concerning risk factors influencing the onset of the disease cannot be tested through this design [3]. One of the reasons is the long duration of the neurodegenerative diseases, thus posing logical challenges. Another could be difficult ethical and logical considerations before exposing not only the healthy study participants but also the researchers to potential risk factors.

The last years have seen emergence of a modern statistical methodology called Mendelian randomization (MR) [4–6]. This methodology uses statistical measures from a handful to dozens of genetic variants, preferably single nucleotide polymorphisms (SNPs), which have been previously studied as markers in context to both causal risk factors and diseases of interest in an observational study setup, and it is believed to provide valid information on existence as well as direction of causation. Alleles at genetic variants are randomly distributed from parents to offspring and are static throughout an individual’s lifetime. An MR study utilizes these naturally randomized proxy markers for the risk factors without the need for measuring risk factors in a population with available data on outcome only to judge causality in an observational study. Such an approach is believed to provide evidence of causality analogous to an RCT. However, it has been also realized over the years that the fruits of this methodology may not be easy to reap. Although it offers an attractive and rapid approach, the methodology comes with number of statistical and theoretical (mainly biological) assumptions which are often impossible to validate [7]. Lack of any consensus in methodologies in the published literature and well-defined steps in an MR workflow add another layer of complexity for interpretation by a common reader. To date, several MR studies exploring causal roles of common biomarkers and other risk factors in some of the neurodegenerative diseases namely AD and various types of dementia have been conducted [8]. Recently, few systematic reviews have also been conducted with a major focus on summarizing the heterogeneity rather than explaining the reliability of clinical interpretations [9–11]. Hence, it is imperative to conduct an integrated systematic review which not only evaluates the quality of studies which have been conducted but also assesses the clinical implications that could be drawn. Furthermore, such a systematic review could also provide direction for future research, specifically careful planning and meaningful interpretations that could be drawn from MR studies on neurodegenerative diseases. Currently, we are witnessing an exponential growth in not only the number of new genome-wide association studies (GWAS) with novel potential risk factors but also the constantly updated meta-GWAS of known risk factors as well as outcomes. In summary, discovering causality through MR approach promises huge opportunities in the coming years provided that we manage the information overload qualitatively and efficiently.

Prior systematic reviews have either focused on methodologies reported in general with limited attention to individual exposure-outcome association or focused on summarizing clinical findings with limited attention to the underlying study design [9–11]. Thus, an integrative approach on studying the influence of MR methodology with respect to specific outcomes and findings is lacking. Furthermore, a comparative approach weighing the reported results by the strength of the MR study design is missing. The proposed systematic review therefore is designed to fill this gap and to help shaping future practices in not only the conduct but also the reporting of MR studies.

### Aims and objectives

Our overarching aim is to inform researchers from diverse backgrounds and enhance productive utilization and meaningful interpretation of MR in the light of existing statistical challenges and gaps in biological knowledge. We plan to follow a critical appraisal approach and accomplish each of the following objectives in a stepwise manner:

1. To assess the methodological quality of MR studies;
2. To assess the results of published data and summarize the clinical interpretations;
3. To evaluate the influence of applying varying MR methodologies on reporting discrepancies in risk factor specific causality.

## Methods and design

The systematic protocol was designed in accordance with the Preferred Reporting Items of Systematic Reviews and Meta-analysis for Protocols (PRISMA-P) 2015 [12]. A completed checklist had been provided as PRISMA-P checklist (Additional file 1).

### Protocol and registration

The protocol was registered on the PROSPERO International Prospective Register of Systematic Reviews (CRD42018091434).

### Study strategy and eligibility criteria

A detailed plan for the conduct of systematic review has been laid down in the form of a schematic flowchart in the Figure. We conducted a pilot study using the search term “Mendelian Randomization” and “Neurodegenerative diseases” in MEDLINE (PubMed) and Web of Science databases. Our pilot study used a paper-based questionnaire comprising a set of 67 questions to extract the relevant information. The questions were designed on the basis of a published MR methodology paper and relevant clinical information required for causal interpretation [13]. All the questions could be classified into the following categories and sub-categories:

1. Stating the hypothesis and identification of datasets: hypothesis, instrument-exposure-outcome triad, individual-level or summary-level data, single-sample or two-sample or sub-sample MR, meta-analyzed datasets;
2. Prioritization of genetic instrument: use of external or internal dataset, functional hypothesis or GWAS based instruments, characteristics of GWAS study;
3. Check for MR assumptions (validation of the genetic instrument): strength, association with confounders, known pleiotropic function of SNPs, presence of population stratification;
4. Estimation of causal effect: ratio of coefficient estimator, two-stage least square, control function estimator, limited information maximum likelihood, inverse-variance weighted, MR-Egger, median weighted method, multivariable MR;
5. Sensitivity analysis: test of heterogeneity, test of endogeneity, leave one SNP out, comparison of MR methods;
6. Additional analyses: power calculation, stratified analysis, check for bi-directionality, use of simulations, use of control MR (positive and negative control MR studies), use of replication cohorts.

Based on the data extraction from our pilot study and frequent consultation between the data extractors, we further refined our questions and finalized a set of 92 questions in an excel sheet format **(**Additional file 2**)**. We further defined the screening strategy for shortlisting publications for subsequent data extraction on the basis of several inclusion and exclusion criteria as listed in Additional file 3.

Furthermore, every publication may describe a single or multiple hypotheses exploring causality using an MR approach, where a hypothesis is defined as a specific combination of exposure (any risk factor), outcome (any neurodegenerative disease), and genetic instrument (one or more SNPs). We treated every hypothesis as a single observation and considered it separately at the time of data extraction.

### Search criteria

One researcher (SG) will conduct the initial search. A literature search of the electronic databases MEDLINE, Embase, Web of Science, Scopus, and databases listed in the Cochrane library will be conducted. The search strategy will include terms related to Mendelian randomization and various types of neurodegenerative diseases. Through the use of Boolean operators, we will search the numerous synonyms and related words as MeSH/Emtree terms (where applicable) and as keywords (for title, abstract, and keyword searches). Specifically, the database searches will be restricted to combinations of an MR term: (“Mendelian Randomization Analysis” OR “Mendelian Randomization” OR “Mendelian Randomisation” OR “Genetic Instrument” OR “Genetic Instrumental” OR “Instrumental variable”) AND a neurodegenerative disease term (“Neurodegenerative Diseases” OR “Neurodegeneration” OR “Neurodegenerative” OR “Dementia” or “Alzheimer disease” or “Alzheimer’s disease” or “Parkinson disease” or “Parkinson’s disease” or “Motor Neurone disease” or “Motor Neuron disease” or “Amyotrophic Lateral Sclerosis” or “Huntington’s disease” or “Huntington’s Chorea” OR “Multiple Sclerosis.”

With respect to MEDLINE, the terms based on MeSH headings will be searched using the [mh] function and other terms will be searched using the [tiab] function to look into title as well as abstract sections of the articles. With respect to Embase, we will further look up MeSH terms in the Emtree Thesaurus to identify additional index terms that will work in Embase. The “ti, ab.” function will be used to search titles and abstracts. With respect to other databases, we will remove the PubMed-specific MeSH terms and replace them using the text search terms and removing the PubMed specific [tiab] field descriptor. A topic search will be adopted for Web of Science search.

**Fig. 1:**
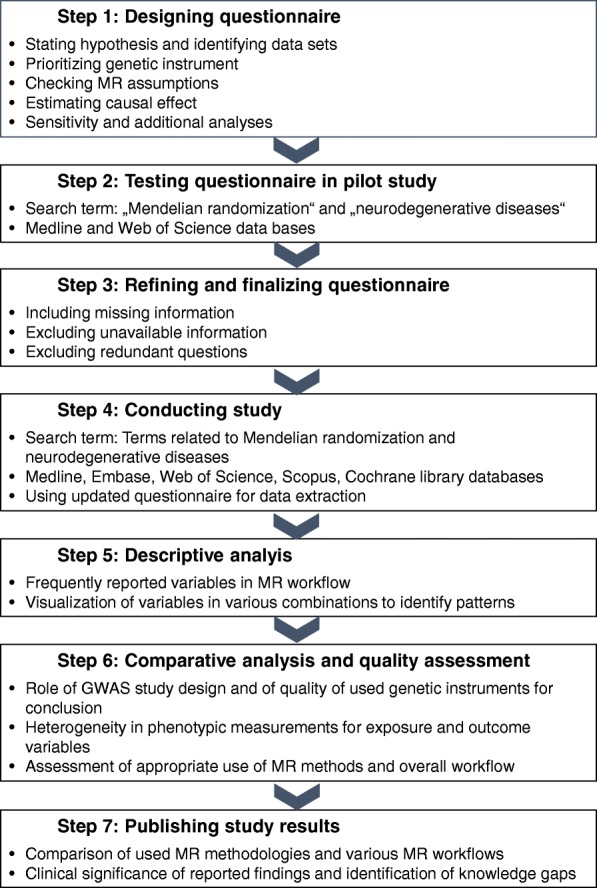
Schematic flow of work plan

All databases will be searched from the earliest dates available (Fig. 1). Two reviewers (SG and IK) will independently screen the titles and abstracts for all the published articles. Non-peer-reviewed literature including conference abstracts and poster presentations will be excluded. Furthermore, references of the shortlisted articles will be screened for any missing articles. This will be followed by data extraction of finalized articles in duplication (SG and IK or SG and FDG). Any discrepancy or disagreement at any stage of article screening and data extraction will be jointly resolved by all the reviewers (SG, IK, and FDG). Since the aim of the current review is also to provide an unbiased evaluation of the available information, no effort was made to contact the authors of the articles. However, if the information was not clearly stated in the MR manuscript, efforts will be made by one reviewer to extract this information from the cited references. As one of the main objectives of the current review is also to judge quality of reporting in the articles, special care will be taken to differentiate between the readily available information from the MR article itself and that extracted from the cited references.

### Data synthesis: narrative and quantitative

The finalized excel datasheet will be imported into an R environment for further descriptive and analytic interpretations [14]. We plan to begin by doing a tabular and graphical summarization of all the variables listed in the Additional file 2.

Based on our pilot study, it was very clear that the probability of finding similar studies to conduct a meta-analysis on the basis of effect estimates is extremely low. One of the main reasons could be the use of updated genetic instruments for exploring causality in the largest available outcome data. However, since most of the latest and largest GWAS studies on exposure and outcome are mostly available in European population, it offers an excellent opportunity to do a narrative assessment. The narrative assessment could help us to draw important conclusions on not only the relative importance of different risk factors for a single outcome but also role of similar markers in different neurodegenerative diseases.

We further plan to compare several extracted variables with respect to their effect on the conclusion of absence or presence of a causal association. Specific interesting investigations include role of study design of GWAS used for prioritizing genetic instrument as well as studying outcome (e.g., discovery or replication cohort), different phenotypic measures of the same outcome (e.g., continuous memory scale vs. categorical AD diagnosis), different methods for generating genetic risk scores (weighted vs. unweighted), different MR methodologies (MR-Egger vs. Inverse-variance weighted vs. Median weighted), and reporting of power calculations.

### Risk of bias across studies

The absence of any existing quality scale or checklist is one of the main motivations behind the current planned systematic review. As stated in the objectives, we plan to develop guidelines suggesting items which should be addressed in an MR article, and we plan to do a quality check scale through this systematic review. We then wish to do a comparative assessment of already published literature in the field of neurodegenerative diseases by applying the scale and thus providing an assessment of risk of bias across the studies in the planned systematic review.

### Dissemination

We plan to disseminate the results on our objectives by peer reviewed publications, presentations at internal project meetings and international scientific conferences. A detailed flowchart showing the study strategy from the inception of idea to design of study to planned conduct of study followed by publication strategy is shown in Fig. 1.

## Discussion

This systematic review is multifaceted with several short- and long-term goals. The short-term goals include a summary of the clinical findings from MR studies in the field of neurodegenerative diseases and the development of MR guidelines applicable to all the diseases. The long-term goals could be the identification of gaps in our knowledge in the field, pooling of existing datasets, and developing a common database of genetic instruments with a clear understanding of GWAS study designs. Although our current systematic review is focused on neurodegenerative diseases, it serves to provide an insight into the current state of MR studies in general. Furthermore, this systematic review along with its the comprehensive data abstraction tool could be directly applied for not only planning an MR study but also understanding the progress made in MR studies irrespective of the outcome under consideration.

Despite several strengths, it is important to emphasize upon some of the limitations of current systematic review. Firstly, we did not use the Delphi expert consensus method to design our questionnaire. Employment of the Delphi method is believed to provide more rational decisions by a group of people on inclusion of specific questions in the questionnaire for judging the quality of studies [15]. It relies on the two main principles of diversity of expertise and independence of decisions by individual team members. Nevertheless, our team of reviewers includes experts from diverse areas of expertise including methodology, statistical genetics, and biology. Secondly, we did not include non-peer-reviewed literature. In addition to non-peer-reviewed findings revealed in conference abstracts and posters, we have also seen the recent emergence of preprint servers including bioRxiv as online publishing platforms for sharing non-peer-reviewed articles [16]. Although non-inclusion of non-peer-reviewed articles could introduce systematic bias in our findings, we decided in favor of peer-reviewed article as peer review process is expected to have major influence on MR methodology including sensitivity analysis. And lastly, it may be argued that we do not contact authors, and this may also introduce bias in our study. However, one of the main objectives of our study was to check the completeness of information in MR articles for readers to judge validity and reliability of results, without the need to contact the corresponding authors. Moreover, due to the comprehensive nature of our systematic review, we believe that it may not be possible for each author to send us information on numerous questions in a reasonable time frame.

In future, our planned comprehensive systematic review will further provide us a platform to assess the quality of the genetic instruments and summarize study designs of underlying GWAS or meta-analysis of GWAS used for extracting genetic instruments for commonly investigated potential risk factors. This will eventually enable us to develop a database of genetic instruments for commonly investigated potential risk factors based on biological knowledge of the functional role as well as unbiased association signals of SNPs constituting the genetic instrument. Such a database is the need of the hour and would help to prevent cherry picking of genetic instruments. And lastly, it will be important to develop guidelines for the conduct and report of an MR study. This would significantly accelerate the development of this exciting field of MR in the right direction to provide an essential tool in clinical decision making. Our results are expected to be publically available in the form of peer-reviewed publications in 2018/2019.

## Additional files

Additional file 1: PRISMA-P checklist. (DOC 81 kb)

Additional file 2: Data abstraction form. (XLSX 23 kb)

Additional file 3: Inclusion and exclusion criteria. (XLSX 9 kb)

## Abbreviations

AD: Alzheimer’s disease
ALS: Amyotrophic lateral sclerosis
GWAS: Genome-wide association study
MR: Mendelian randomization
MS: Multiple sclerosis
PD: Parkinson’s disease
PRISMA-P: Preferred Reporting Items for Systematic Reviews and Meta-analysis Protocols
RCT: Randomized controlled trial
SMR: Summary-data-based MR
SNP: Single nucleotide polymorphism
